# Association of psychological and financial distress with physical activity among Hispanic/Latino populations during the COVID-19 lockdowns in the United States

**DOI:** 10.1080/29944694.2026.2712532

**Published:** 2026-08-07

**Authors:** Heramb Gupta, Matthew A. Allison, Yasmin Mossavar-Rahmani, Krista M. Perreira, Linda C. Gallo, Elizabeth C. Oelsner, Kelly R. Evenson, Martha Daviglus, Olga Garcia-Bedoya, Daniela Sotres-Alvarez, Carmen R. Isasi, Robert Kaplan, Kunihiro Matsushita, Jennifer A. Schrack, Pablo Martinez-Amezcua

**Affiliations:** a Department of Epidemiology, Johns Hopkins Bloomberg School of Public Health, Baltimore, MD, USA; b Division of Preventive Medicine, University of California, San Diego School of Medicine, San Diego, CA, USA; c Department of Epidemiology & Population Health, Albert Einstein College of Medicine, Bronx, NY, USA; d Department of Social Medicine, School of Medicine, UNC Chapel Hill, Chapel Hill, NC, USA; e Department of Psychology, College of Sciences, San Diego State University, San Diego, CA, USA; f Division of General Medicine, Department of Medicine, Columbia University Irving Medical Center, New York City, NY, USA; g Department of Epidemiology, Gillings School of Global Public Health, UNC Chapel Hill, Chapel Hill, NC, USA; h Institute for Minority Health Research, School of Medicine, University of Illinois Chicago, Chicago, IL, USA; i Department of Biostatistics, Gillings School of Global Public Health, UNC Chapel Hill, Chapel Hill, NC, USA; j Division of Public Health Sciences, Fred Hutchinson Cancer Research Center, Seattle, WA, USA

**Keywords:** Physical activity, COVID-19, mental health

## Abstract

We examined the links between COVID-19-related concerns, psychological distress, material hardship, and physical activity (PA) among Hispanic/Latino adults in the U.S. during the pandemic. We conducted a cross-sectional analysis of data from 243 participants in the Hispanic Community Health Study/Study of Latinos (HCHS/SOL), collected between January and July 2021. Participants underwent phone interviews assessing COVID-19 concerns, psychological distress via the PHQ-4, and material hardship. PA was measured with wrist-worn accelerometers over seven days, tracking total activity counts/day, active minutes/day, and activity fragmentation (active-to-sedentary transition probability, ASTP). We used regression models adjusted for demographic, behavioral, and health covariates. Participants (mean age = 62; 67% female) showed high psychological distress (63%) and material hardship (33%), with 39% reporting COVID-19 concerns. Concerns were linked to lower total activity counts (β = −231, 95% CI: [−459, −3]) and active minutes/day (β = −42.1, 95% CI: [−76.7, −7.4]) in adjusted models. While ASTP was higher among individuals with COVID-19 concerns, this association diminished after full adjustment. No significant links were found between material hardship or distress and PA. COVID-19-related concerns were associated with reduced and fragmented physical activity, suggesting that addressing psychosocial stressors may help improve health behaviors in this population during public health emergencies.

## Introduction/background

Since December 2019, COVID-19 has spread across the world. During the initial phases, many preventative actions were taken, including lockdowns characterized by the closure of educational institutions and commercial spaces, social distancing measures, and travel restrictions—the significant restriction of life space mobility limited physical activity (PA) and increased sedentary behavior (Wilke et al., 2022). Additionally, the pandemic had a considerable impact on mental health, namely increasing levels of anxiety, depression, and stress (Pfeifer et al., 2021). Moreover, shutdowns led to financial instability, material hardships (difficulty paying for basic needs), and unemployment (Liu et al., 2022; Rose, 2021).

Marginalized groups bore a disproportionate burden of the pandemic’s impact. In the United States (US), counties with a higher proportion of Hispanics/Latinos, Asians, and Blacks had higher COVID-19 rates (Abedi et al., 2021). The disparities in living and working conditions left these minoritized racial and ethnic groups more vulnerable to COVID-19 during the lockdowns. Hispanics/Latinos faced greater economic challenges compared to other demographic groups and had a higher risk of COVID-19 hospitalizations and mortality than non-Hispanic/Latino Whites (Center for American Progress, 2021).

PA had several positive effects during the COVID-19 pandemic, including improved mental health and enhanced recovery after infection (Yang et al., 2022). However, little is known about the influence of concerns related to COVID-19, psychological factors, and material hardships on PA among adults of Hispanic/Latino heritage. To address this gap, we investigated the associations between these stressors and PA among Hispanics/Latinos living in the US during the pandemic.

## Methods

### Study design and population

The Hispanic Community Health Study/Study of Latinos (HCHS/SOL) is an ongoing cohort study of the health of the Hispanic/Latino population in four US cities—The Bronx, NY; Chicago, IL; Miami, FL; and San Diego, CA. During the COVID-19 pandemic, HCHS/SOL became part of the Collaborative Cohort of Cohorts for COVID-19 Research (C4R) to understand the acute and long-term impacts of COVID-19 on health (Oelsner et al., 2022). This study aims to analyze the relationship between COVID-19, psychosocial and financial, and PA. Data were collected regarding participants’ COVID-19-related experiences using phone questionnaires between January 26, 2021, and July 27, 2021, and PA using wrist accelerometry between February 02, 2021, and July 27, 2021. Two hundred eighty-three participants were interviewed for the study and asked to wear an accelerometer for seven days. For the current analysis, we excluded 37 participants with missing accelerometer data or <3 valid accelerometer wear days (valid = wear ≥90% of the day) (Schrack et al., 2014) and 3 participants without COVID-19 data, leaving an analytic sample of 243 participants. The study was conducted with Institutional Review Board approval from each of the institutions involved in the study.

### Assessment of physical activity

Participants were instructed to wear a triaxial wrist-worn accelerometer (ActiGraph Link, ActiGraph, Pensacola, FL) continuously on their non-dominant wrist for seven days (except for water submersion >30 minutes). The accelerometers were sent and returned via US mail. The following metrics were derived: 1) total activity counts/day (TAC, calculated as the mean vector magnitude across the three axes), measuring volume and intensity; 2) active minutes/day (time above the threshold of 1,853 counts/minute), measuring volume only; and 3) activity fragmentation (active to sedentary transition probability [ASTP], or the reciprocal of the mean active-bout duration) (Schrack et al., 2019). Higher ASTP values indicated increased breaking-up of active bouts.

### Assessment of COVID-19 related experiences

We assessed the COVID-19-related experiences of participants through phone interviews that included the following questions.

### COVID-19 concerns

Participants were asked to rate their concern regarding COVID-19 or its associated illness on a scale from 1 (not at all concerned) to 5 (extremely concerned). For analysis, we categorized participants based on the distribution of the answers into low (≤3) and high levels of concern (>3).

### Psychological distress

Psychological distress over the last 2 weeks was measured using the PHQ-4 depression and anxiety screener. There were two items for depression (loss of interest, depressed mood) and two for anxiety (nervousness, worry). Each item is scored from 0 to 3 based on the frequency of symptoms (not at all-nearly every day), and the sum of scores (range 0–12) was used to identify psychological distress (sum score ≥3).

### Material hardship

Material hardship included yes/no questions about accessibility to basic amenities, rent or mortgage payments, and food scarcity. This variable was dichotomized as any vs. no hardship.

### Covariates of interest

Demographic covariates included age, sex, education, and region of birth (categorized into Mexico, Cuba, Dominican Republic, Puerto Rico, North America, South America, Central America, and others). Health covariates encompassed body mass index (BMI; measured in HCHS/SOL previous visit: 2014–17), diet quality (Alternative Healthy Eating Index score, 2008–11), alcohol consumption (2014–17 to 2021), smoking status, diabetes, hypertension, asthma, and LDL cholesterol levels (2021). Participants were asked if they were unemployed or employed (part or full-time) before January 1, 2020, and after March 1, 2020, and this change of employment was used as a covariate.

### Statistical analysis

We used linear regression models that account for geographical clusters, to investigate the association between COVID-19-related psychosocial experiences and daily PA. We built separate models for COVID-19-related concerns, psychological distress, and material hardship as independent variables. Model 1 was adjusted for age, sex, site, education, and accelerometer wear time. For Model 2, we further adjusted for change in employment status, alcohol consumption, smoking status, diet quality, country of origin, BMI, asthma, diabetes, hypertension, and dyslipidemia. As a regression diagnostic test, we estimated the variance inflation factor (VIF) to assess collinearity among the independent variables in a separate model that included all three exposures simultaneously and covariates in model 2. For sensitivity analyses, we accounted for COVID-19 cases in participants’ counties (from the CDC website) from the previous month, as well as the lag between accelerometer wear and questionnaire response dates.

## Results

Of 243 participants (mean age = 62 ± 9, 67% female), 39% expressed COVID-19 concerns, 21.4% had psychological distress, and 33% faced material hardship (Table 1). Most participants were from the San Diego site, and had high school or higher educational attainment. The majority reported no smoking and 43% reported current alcohol use. Chronic conditions were common, especially hypertension (51.0%) and hypercholesterolemia (57.6%), while asthma (14.8%) and diabetes (22.6%) were less frequent.

Participants with COVID-19 concerns had lower total activity counts (Table 2: β= −231, 95% CI: [−459, −3] in Model 2) and fewer active minutes per day (β=−42.1 min/day, 95% CI: [−76.7, −7.4]). Additionally, COVID-19 concerns were associated with a higher Active to Sedentary Transition Probability (ASTP) in Model 1 (β = 1.76, 95% CI: [0.16, 3.36]), though this association was attenuated in Model 2. No significant associations were observed between material hardship or psychological distress and physical activity metrics. Our VIF analysis suggested that collinearity was not a concern with VIF values <2 for all variables. Sensitivity analyses yielded similar results.

## Discussion

We investigated the association of COVID-19-related experiences with PA levels among Hispanics/Latinos in four diverse US cities during the pandemic. We found that greater COVID-19 concerns were associated with lower and more fragmented activity. Our study adds to the understanding of the complex relationship between psychosocial factors, which surfaced with COVID-19 lockdowns, and PA in Hispanics in US cities (Isasi et al., 2023).

In the US context during the COVID-19 lockdowns, our findings of lower activity among those who reported greater COVID-19 concerns may reflect risk-averse attitudes and greater compliance with reduced mobility mandates among Hispanics/Latinos, a population disproportionately affected by structural inequities such as multigenerational households and the overrepresentation of essential workers, hindering the ability to isolate (Hasson et al., 2022; Patel et al., 2023). Moreover, the closure of gyms and exercise group programs may have intensified existing barriers to physical activity, particularly in less walkable neighborhoods, which are known to influence activity among Hispanics (Carlson et al., 2022). Our findings for COVID-19 concerns were consistent across models for activity outcomes, except for attenuation and a nonsignificant finding for ASTP after adjustment for variables in model 2. This could be due to differences in ASTP, an activity measure more closely related to endurance capacity, between low and high COVID-19 concern groups, being explained by factors included in model 2, such as comorbidities and BMI

The fact that psychological distress and material hardship were not associated with physical activity outcomes, but COVID-19 concerns were, suggests that pandemic-specific risk perceptions may have been more proximal to behaviors than emotional or financial strains. Psychological distress may have affected physical activity only minimally or indirectly, as our nonsignificant findings suggest. For material hardship, we found no statistically significant findings, but the coefficients indicated that greater hardship was associated with greater activity. People with greater hardship may have more complex activity patterns, with greater occupational activity (if they continued or started working in essential positions to maintain a source of income) and lower leisure-time activity (due to restrictions or fatigue induced by job demands).

Overall, our findings may inform targeted and culturally relevant interventions to promote mental well-being and PA, especially among marginalized communities. Future studies should expand the scope to include rural populations to advance our understanding of the multifaceted factors influencing health behaviors during health crises.

The study’s strengths include the use of accelerometry for objective activity measurement, a well-characterized study population, and geographic diversity. Limitations include the cross-sectional design, temporal mismatch in the measurement of some covariates (e.g., BMI), the absence of rural populations in the sample, the lack of information on how vaccines and new virus variants may have affected PA, and potential variations in the interpretation of the mental health questionnaire (Likert scales for COVID-concern, which have inherent psychometric limitations), possibly leading to measurement error in our exposures.

Future public health efforts must consider the specific psychosocial and economic challenges faced by marginalized and vulnerable groups during public health crises to prevent further adverse effects and monitor efforts to address or intervene in associated health outcomes.

Greater COVID-19 concerns were associated with lower and more fragmented physical activity among Hispanics/Latinos in four U.S. cities. These findings highlight the need for targeted public health strategies that support physical activity and mental well-being during health crises, especially in marginalized communities.

## Figures and Tables

**Table 1.** Characteristics of the study population.

Characteristic	Total (N = 243)

COVID-19 concern, n (%)	94 (38.68)
Material hardship, n (%)	80 (32.92)
Psychological distress, n (%)	52 (21.40)
Age, mean (SD)	62.29 (9.74)
Females, n(%)	162 (66.67)
Study site, n (%)	
Bronx	61 (25.10)
Chicago	47 (19.34)
Miami	62 (25.51)
San Diego	73 (30.04)
Education, n (%)	
< High School	59 (24.28)
High School	94 (38.68)
>High School	85 (34.98)
Employment change, n (%)	
No change, Unemployed	127 (52.26)
No change, Part time	16 (6.58)
Reduced, Part time	32 (13.17)
No change, Full time	24 (9.88)
Reduced, Full time	43 (17.70)
Alcohol use, n (%)	
Never	87 (35.80)
Current	103 (42.39)
Former	40 (16.46)
Missing	13 (5.35)
Smoking status, n (%)	
Never	136 (55.97)
Current	65 (26.75)
Former	41 (16.87)
AHEI score, mean (SD)	50.22 (7.51)
BMI, mean (SD)	29.42 (5.44)
Asthma, n (%)	36 (14.81)
Diabetes, n (%)	55 (22.63)
Hypertension, n (%)	124 (51.03)
Cholesterol, n (%)	140 (57.61)

**Table 2.** Association between psychosocial and financial distress and accelerometer-measured physical activity (N = 243).

Exposures	Accelerometry-assessed physical activity outcomes					
	Total Activity Counts (Thousands/day)		Active Time (Minutes/day)		Active-to-Sedentary Transition Probability (%)	
	Model 1 β (95% CI)	Model 2 β (95% CI)	Model 1 β (95% CI)	Model 2 β (95% CI)	Model 1 β (95% CI)	Model 2 β (95% CI)

COVID-19-related concerns	**−280** **(−501, −58)**	**−231** **(−459, −3)**	**−50.6** **(−83.7, −17.5)**	**−42.1** **(−76.7, −7.4)**	**1.76** **(0.16, 3.36)**	1.29 (−0.24, 2.84)
Material hardship	51 (−174, 276)	50 (−162, 262)	−3.4 (−31.5, 38.4)	5.7 (−26.2, 37.7)	−0.31 (−2.01, 1.39)	−0.46 (−2.03, 1.12)
Psychological distress	−193 (−434, 50)	−111 (−344, 121)	−30.1 (−69.0, 8.8)	−19.0 (−58.4, 20.5)	2.11 (−0.01, 4.25)	1.54 (−0.63, 3.70)

Model 1: adjusted for age, sex, site, education, and accelerometer wear time.

Model 2: further adjusted for employment status change, alcohol consumption, smoking status, alternative healthy eating index, country of origin, body mass index, asthma, diabetes, hypertension, and dyslipidemia.

Abbreviations: ASTP - Active-to-Sedentary Transition Probability (Activity Fragmentation); CI – confidence interval.

* Bolded entries represent a significant association (*p* < 0.05).

## Data Availability

Data are publicly available through NIH repositories.
